# Lay responder naloxone access and Good Samaritan law compliance: postcard survey results from 20 Indiana counties

**DOI:** 10.1186/s12954-018-0226-x

**Published:** 2018-04-06

**Authors:** Dennis P. Watson, Bradley Ray, Lisa Robison, Philip Huynh, Emily Sightes, La Shea Walker, Krista Brucker, Joan Duwve

**Affiliations:** 1 Department of Social and Behavioral Sciences, Center for Health Engagement and Equity Research, Indiana University Richard M. Fairbanks School of Public Health, Indiana University-Purdue University Indianapolis, 1050 Wishard Blvd, Indianapolis, IN 46202 USA; 2 School of Public and Environmental Affairs, Indiana University-Purdue University Indianapolis, 801 W. Michigan St, Indianapolis, IN 46202 USA; 3 Department of Emergency Medicine, Indiana University School of Medicine, Indiana University School of Medicine, 3930 Georgetown Rd, Indianapolis, IN 46254 USA; 4 Department of Health Policy and Management and the Center for Public Health Practice, Indiana University Richard M. Fairbanks School of Public Health, Indiana University-Purdue University Indianapolis, 1050 Wishard Blvd, Indianapolis, IN 46202 USA

**Keywords:** Naloxone, Narcan, Opioid, Overdose prevention, Lay responder, Good Samaritan

## Abstract

**Background:**

To reduce fatal drug overdoses, two approaches many states have followed is to pass laws expanding naloxone access and Good Samaritan protections for lay persons with high likelihood to respond to an opioid overdose. Most prior research has examined attitudes and knowledge among lay responders in large metropolitan areas who actively use illicit substances. The present study addresses current gaps in knowledge related to this issue through an analysis of data collected from a broader group of lay responders who received naloxone kits from 20 local health departments across Indiana.

**Methods:**

Postcard surveys were included inside naloxone kits distributed in 20 Indiana counties, for which 217 returned cards indicated the person completing it was a lay responder. The survey captured demographic information and experiences with overdose, including the use of 911 and knowledge about Good Samaritan protections.

**Results:**

Few respondents had administered naloxone before, but approximately one third had witnessed a prior overdose and the majority knew someone who had died from one. Those who knew someone who had overdosed were more likely to have obtained naloxone for someone other than themselves. Also, persons with knowledge of Good Samaritan protections or who had previously used naloxone were significantly more likely to have indicated calling 911 at the scene of a previously witnessed overdose. Primary reasons for not calling 911 included fear of the police and the person who overdosed waking up on their own.

**Conclusions:**

Knowing someone who has had a fatal or non-fatal overdose appears to be a strong motivating factor for obtaining naloxone. Clarifying and strengthening Good Samaritan protections, educating lay persons about these protections, and working to improve police interactions with the public when they are called to an overdose scene are likely to improve implementation and outcomes of naloxone distribution and opioid-related Good Samaritan laws.

## Background

Opioid-related overdoses are now the leading cause of preventable death in the USA [1–3]. In an effort to quell the rising tide of opioid overdose deaths, every US state and the District of Columbia have passed laws improving both first responder and lay responder—i.e., regular citizens who are in a position to respond to an opioid overdose—access to naloxone (also known by the brand name Narcan), the opioid antagonist used to reverse the symptoms of an opioid overdose [4, 5]. Illicit opioids, including natural opioids mixed with synthetic analogs and pure synthetic analogs, are extremely potent and can require multiple doses of naloxone to counteract. Therefore, it is recommended that administration of naloxone be coupled with appropriate emergency medical care in case follow-up supportive care and additional doses of naloxone are needed [6, 7]. However, many lay responders do not call 911 [8–10] because they fear legal repercussions (e.g., arrest, loss of public housing, or benefits) [8, 9, 11–20]. It is for this reason that, as of July 2017, 40 states have passed Good Samaritan laws/protections that safeguard individuals who report an overdose in “good faith” from certain criminal sanctions [21], with specific protections varying by state.

Demonstrated effectiveness of naloxone education and distribution programs supports the rationale behind naloxone access and Good Samaritan laws that bystanders should not fear legal repercussions for offering assistance to someone injured or otherwise incapacitated [22–25]. Moreover, public and professional attitudes generally support these laws [26–28]. Yet, studies continue to demonstrate 911 is frequently not called despite the presence of a Good Samaritan law due to either a lack of awareness or continued fear of criminal liability [20, 29–31]. These studies have largely focused on lay responders who are themselves opioid users (primarily injection opioid users), and each study is generally focused on a population from a limited urban geographic area (e.g., a single city or metropolitan area). The current study adds to this literature by examining data from a naloxone distribution program operating in 20 counties across Indiana that provided naloxone to lay responders. Each county’s local health department was provided with the naloxone kits through a grant mechanism administered by the Indiana State Department of Health. Our results focus on the variation in experiences between those who obtained naloxone for someone else and those who obtained it for themselves as well as reasons for not having previously called 911 during an overdose.

## Methods

Postcard surveys were included inside naloxone kits distributed in 20 Indiana counties. Postcard surveys can be useful for obtaining survey data from research subjects who lack time to fill out long questionnaires and/or may perceive such questionnaires as burdensome [32].

The number of kits distributed in each county ranged from 10 to 400 (M = 94; Mdn = 50). According to the US Census [33], county populations ranged from 23,087 to 903,393 (M = 103,641; Mdn = 37,800), and all populations were between 87 and 98% White, except for the largest county, which was 63% White. Only eight counties could be considered urban (or to have urban areas) in that they had a sizable number of their population living in a metropolitan area [34].

### Respondents

Survey respondents are individuals who obtained a naloxone kit from their local health department. A total of 3474 kits were provided to health departments. Of these, approximately 1767 kits were distributed 1 year later (based on kit distribution information supplied by the health departments), for which we received 767 completed postcards (an estimated response rate of about 43%). Researchers were originally informed that the kits were only being made available to lay responders; however, our data demonstrated a number of professional first responders and other health care professionals were accessing the kits (likely due to limited availability through their employers). Our analysis in this paper focuses on lay responders (i.e., survey respondents who indicated they obtained the naloxone kit for themselves, a family member, and/or friend).

### Measures

Each postcard requested demographic information, including age, zip code, gender, and race/ethnicity, and asked respondents to indicate who they obtained the kit for (e.g., themselves, a family member, a friend, a client or patient, or “other” reason). The postcard requested information on the respondent’s experiences with opioid overdose, including if (1) they know anyone who has experienced a fatal or non-fatal overdose, (2) how many overdoses they have witnessed, and (3) how recently (i.e., within the past month, last year, or more than a year ago) they have witnessed an overdose. As a follow-up question, respondents were asked whether 911 was called at the scene of the last overdose witnessed and, if not, the reason why 911 was not called (i.e., worried about police, person woke up on their own, no phone available, they did not want to upset the victim). Lastly, respondents were asked if they are aware of the law that protects witnesses from drug charges when 911 is called to the scene of an overdose and if they have previously administered naloxone prior to receiving the kit.

### Procedures

The back side of the postcard included instructions describing the research purpose, the confidential nature of the study, when to complete the postcard survey, and how to return the postcard. All postcards were pre-addressed and included pre-paid postage, and the participant was asked to return the card via any US mail postbox. Each health department was sent an information sheet about the survey that asked them to inform lay responders of the postcard at the time the kit was provided. Health department staff were also requested to encourage lay responders to complete the survey after receiving the kit. Lay responders were not required to complete the postcard. Researchers provided each health department with a drop box where respondents could place the card in if they chose to fill it out before leaving; health department staff were instructed to regularly empty this box into the US mail. Researchers followed up with a staff member at each health department to ensure the information was received and understood. Naloxone kits were distributed between September 9, 2016, and February 9, 2017.

### Analysis

To ensure the analysis focused on lay responders, all individuals who indicated obtaining the kit for a client or patient or who did not indicate who they obtained the kit for were excluded. Of the 767 postcards returned, 235 were identified as being completed by lay responders (the rest being first responders or were unidentifiable due to missing data); however, after excluding cases with missing variables (*n* = 18; 7.7%), we were left with a final sample of 217 cases. Of these cases, an additional 5.5% (*n* = 12) had data imputed. We only imputed data if it could logically be discerned from the respondent’s answers to previous questions. For instance, if respondents indicated they had never witnessed an overdose, we imputed data for subsequent unanswered overdose questions as “I haven’t seen an overdose.” Descriptive and correlation statistics were used to examine and describe the results of the postcard data. Statistical tests that were performed include t-tests and chi-square (*χ*2) difference of proportion tests. The level of significance was *α* = 0.05, with all *p* values lower than that value considered statistically significant.

## Results

The sample’s demographic characteristics are displayed in Table 1. The average age of respondents was 47.2 years (SD = 14.8), and over two thirds (*n* = 151; 69.5%) were over age 40. Slightly more than half (55.3%) were female, and the majority were White (88.0%). Among those coded as non-White, 76.9% (*n* = 20) were Black, 0.1% (*n* = 2) were Asian, 0.1% (*n* = 3) were American Indian/Alaskan Native, and 0.2% (*n* = 5) indicated multiple race/ethnicity categories.

**Table 1:** Sample demographics (*N* = 217)

	M (SD)
Age	47.2 (14.8)
	*N* (%)
Sex	
Female	120 (55.3)
Male	97 (44.7)
Race/minority status	
Non-White	26 (12.0)
White	191 (88.0)
Age categories	
Under 19	5 (2.3)
20–29	27 (12.4)
30–39	34 (15.7)
40–49	50 (23.0)
50–59	54 (24.9)
60–69	32 (14.7)
70 and over	15 (6.9)

Table 2 displays the items measuring past experiences with naloxone and overdose and knowledge regarding Good Samaritan protections. Few respondents (7.4%) had used naloxone before; however, approximately two thirds (62.2%) knew someone who had overdosed from opioids, and nearly half (47.9%) knew someone who had died from an opioid overdose. Over one third (*n* = 75; 34.6%) had directly witnessed an opioid overdose; among these cases most witnessed only one. Among those who reported having witnessed an overdose (*n* = 75), 85.3% had their most recent experience sometime in the past year. When asked if someone called 911 at this most recent overdose experience, 80.0% reported they had. Among those who did not call 911, the most common reasons were that they were worried about the police (*n* = 6), the person woke up on their own (*n* = 5), the person was transported to a hospital (*n* = 3), or an unspecified reason (*n* = 4).

**Table 2:** Experience with overdose and knowledge of Good Samaritan protections (*N* = 217)

	*N* (%) Yes
Have you used naloxone before? (Yes)	16 (7.4)
Do you know anyone who has overdosed from opioids? (Yes)	135 (62.2)
Do you know anyone who has died from an opioid overdose? (Yes)	104 (47.9)
Are you aware of the law that protects overdose victims and witnesses from drug charges when 911 is called to the scene of an overdose?	168 (77.4)
	*N* (%)
About how many opioid overdoses have you witnessed?	
None	142 (65.4)
1 overdose	30 (13.8)
2 overdoses	17 (7.8)
3 overdoses	10 (4.6)
4 overdoses	6 (2.8)
5 overdoses	6 (2.8)
6 or more overdoses	6 (2.8)
When was the last time you witnessed an opioid overdose?	
Within the past month	33 (44.0)
Within the past 12 months	31 (41.3)
More than a year ago	11 (14.7)
At the last opioid overdose scene you witnessed, did someone call 911?	
Yes	60 (80.0)
No	15 (20.0)

### Intended naloxone recipient

Next explored were variations in prior experiences and knowledge of Indiana’s Good Samaritan protections by who the respondent indicated they were getting the naloxone kit for. For this, two categories of lay responder were created based on the intended recipient of the naloxone: (1) those indicating they obtained the kit for themselves (i.e., marked “self” on the survey) and (2) those who indicated they obtained the kit for others (i.e., marked “family” or “friend” on the survey). Those who indicated obtaining the kit for both self *and* others, which were only three cases (1.4%), were placed in the “other” category. This resulted in 38.7% (*n* = 84) of the respondents being placed into the “self” category and 61.3% (*n* = 133) in the “other” category.

Differences in demographics and prior experiences based on whom the respondent indicated they were obtaining the naloxone for were examined. Those who indicated they obtained naloxone for themselves were significantly younger than those who obtained it for someone else (43.1 and 49.7 years, respectively; *t* = 3.26, *p* = .001) and were also more likely to be non-White (17.9 and 8.3%; *χ*^2^ = 4.48, *p* = .034). There were no significant differences by gender, though a greater portion of females reported obtaining naloxone for others than males (58.6 and 41.4%, respectively; *p = .*212), and among those who obtained naloxone for themselves, gender was evenly split (*n* = 42; 50.0%; *p = .*212) between males and females.

**Fig. 1:**
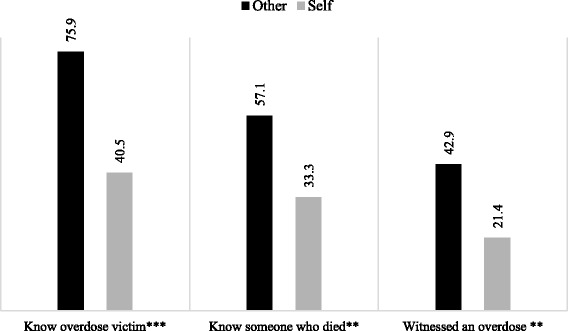
Prior overdose experience and intended naloxone recipient (*N* = 217). **p < .01, ***p < .001

In looking at prior experiences, those who indicated obtaining naloxone for others were significantly more likely to know someone who had overdosed (75.9 vs. 40.5%; *χ*^2^ = 27.54, *p* < .000), know someone who had died of an overdose (57.1 vs. 33.3%; *χ*^2^ = 11.69, *p* = .001), or have witnessed an overdose (42.9 vs. 21.4%; *χ*^2^ = 10.45, *p* = .001) than those who obtained naloxone for themselves (see Fig. 1). Among those who witnessed an overdose, there was no difference in how recently they had seen the overdose or whether they had called 911 at the scene of the overdose by whom they obtained the naloxone for, nor were there any differences in knowledge about Good Samaritan protections and whom the naloxone was obtained for.

### Calling 911 and knowledge of Good Samaritan laws

**Fig. 2:**
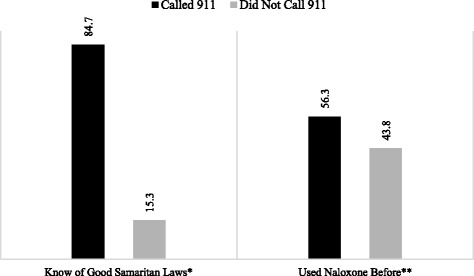
Knowledge of Good Samaritan protections, prior naloxone use, and calling 911 (*n* = 75). *p < .05, **p < .01

A separate analysis was conducted on the subsample of respondents who indicated they had called or not called 911 at the scene of the last overdose they witnessed (*n* = 75) to understand if there was any association between this behavior and (a) respondent demographics, (b) prior experiences with naloxone and overdose, or (c) knowledge of Good Samaritan protections. There was no variation by age, race/ethnicity, or gender, nor were there any differences in whether the respondent knew someone who had died of an overdose. However, as demonstrated in Fig. 2, those with knowledge of Good Samaritan protections were significantly more likely to have called 911 (84.7 vs. 15.3%; *χ*^2^ = 3.89, *p* = .048), and those who had used naloxone before were significantly more likely to have called 911 than those did not (56.3 vs. 86.4%; *χ*^2^ = 7.17, *p* = .007).

## Discussion

Findings from this study are unique in that they focus on responses of the broader lay responder population (rather than just current opioid users) and included a more geographically heterogeneous population than prior work on this topic that we are aware of. Our study revealed that few survey respondents had administered naloxone, but a large portion indicated having witnessed an overdose or knowing someone who had died from an overdose. Furthermore, these experiences were reported at significantly higher rates among those who indicated obtaining naloxone for someone other than themselves. These findings are consistent with studies showing that witnessing an overdose or knowing someone who died from an overdose is potentially a strong motivator for first time naloxone access [35]. However, a better understanding of the broader lay responder population’s awareness and motivations for obtaining naloxone could greatly benefit prevention efforts.

Findings from this study also suggest that among those who had witnessed an overdose, knowledge of the Good Samaritan protections and prior naloxone administration were significantly associated with having called 911. This is consistent with Jakubowski et al. [36] who found correct knowledge of a Good Samaritan law was associated with calling 911 among individuals trained in naloxone administration. Also consistent with previous research, we found that among those who did not call 911, concerns about the police were an underlying reason [8, 9, 11–20], with a secondary reason being that the person woke up on their own. As others have suggested, this finding could indicate an overall lack of trust of police or past negative personal experiences with law enforcement [27, 36–38]. Indeed, some respondents in our sample wrote messages in the margins of the postcard indicating they had heard of others being mistreated despite the law or they believe the police would begin watching their home or “stalking” them even if they did not arrest them at the time of the overdose.

More than three quarters (77.4%) of respondents indicated knowledge of Indiana’s Good Samaritan protections: 76.2% among those obtained naloxone for themselves and 78.2% among those obtained it for someone else. Without a baseline comparison, it is difficult to know if this is high or low, though it is likely high given respondents self-selected to obtain naloxone, with many of them obtaining it for someone other than themselves. Certainly, future research is needed to understand general public knowledge around Indiana’s opioid Good Samaritan protections (and Good Samaritan laws in general), but it is also possible there is general confusion around the legislation, which provides some criminal, as well as civil, protections to lay persons administering naloxone at the scene of an overdose [39, 40]. However, these protections are not comprehensive, as they do not extend to the person who overdosed, and it is unclear to what extent they cover witnesses at the scene of the overdose who did not administer naloxone and/or initiate the 911 call. Furthermore, those protections offered to the lay responder are limited in that they only cover criminal liability for possession of drugs or paraphernalia, making it less likely someone would call if they were at risk for other offenses (e.g., violating parole, having a current warrant for arrest issued, or public intoxication). The limitations of Good Samaritan laws are a noted problem across the USA [4, 6, 21, 41], and strengthening protections offered to lay responders and extending them to the overdose survivors and all witnesses at the scene have potential for improving compliance.

Related to lay responder fears concerning police, an even more recent development in Indiana that has potential to seriously weaken the intended results of the state’s Good Samaritan protections is the passage of a new drug-induced homicide law passed in March 2018 [42]. Drug-induced homicide laws exist in 20 other states, and they raise the penalties associated with dealing drugs should said drugs result in an overdose death [43]. Indiana’s law would raise the penalties associated with opioid dealing to a Level 1 Felony and is broad enough to include any exchange of drugs, regardless of a monetary transaction, to be considered dealing (e.g., sharing with friends or a loved one). Despite an increased implementation of drug-induced homicide legislation, there is currently no evidence supporting the effectiveness of such laws. Indeed, previous research has demonstrated increasing criminal sanctions does not result in less drug demand or sales (see [43–45]). Lay responder awareness of these could result in less willingness to call emergency responders due to greater fear of criminal prosecution (for themselves and/or others present at the overdose scene), thus increasing the risk of overdose death.

While the findings in this study offer insight into the motivations, attitudes, and experiences of lay persons who obtain naloxone, it is not without limitations. Most obvious are limits on information that could be gathered (given the room available on the postcard instrument) and our response rate. However, a strength of the postcard method is that more people likely responded than would have if a more time-consuming survey approach had been used [32], and the response rate is higher than expected considering members of the public who access naloxone from an anonymous, government-run distribution program are unlikely to want to participate in data collection activities, no matter how streamlined they might be. Additionally, inconsistent distribution numbers from health departments required us to estimate the response rate based on information available. This was a reality we had to deal with when working with local health departments, as they are often understaffed and have limited resources, which can affect reporting. It cannot be assured that those indicating they obtained naloxone for someone other than themselves are not also opioid users or whether their knowledge about the existence of Good Samaritan protections is consistent with the facts regarding these laws. However, despite these limitations, this study represents a wider lay responder population than has been examined in similar studies, and our multi-county sample is broader than those included in most previous naloxone distribution research.

## Conclusions

In closing, the results of this study point to prior experience as an opioid overdose witness or knowing someone who died of an opioid overdose as potential motivators for naloxone access among lay responders. Results also demonstrate reluctance among some lay responders to call 911 at an overdose scene despite knowledge of Good Samaritan protections. Additional research is needed to better understand factors motivating naloxone access and compliance with opioid Good Samaritan laws among lay responders who are not themselves opioid users. Finally, efforts to improve implementation of naloxone access and Good Samaritan laws could be strengthened through more intensive public education and efforts to improve interactions between police and people who use illicit opioids.
